# Zinc finger protein ZFP36L1 promotes osteoblastic differentiation but represses adipogenic differentiation of mouse multipotent cells

**DOI:** 10.18632/oncotarget.15246

**Published:** 2017-02-09

**Authors:** Kuo-Yun Tseng, Yi-Hsuan Chen, Shankung Lin

**Affiliations:** ^1^ Institute of Cellular and System Medicine, National Health Research Institutes, Zhunan Town, Miaoli, Taiwan, Republic of China; ^2^ Graduate Institute of Basic Medical Science, China Medical University, Taichung, Taiwan, Republic of China

**Keywords:** ZFP36L1, osteoporosis, aging-related bone loss, osteoblastogenesis, adipogenesis, Gerotarget

## Abstract

Zinc finger protein 36, C3H type-like 1 (ZFP36L1) is a member of the tristetraprolin (TTP) family and its role in the aging-related bone loss is currently unknown. We present evidence that ZFP36L1 expression in rat femurs and bone marrow mesenchymal stem cells (bmMSCs) was down-regulated with aging. ZFP36L1 knockdown decreased osteoblastic differentiation of MC3T3-E1 and C3H10T1/2 cells, and increased adipogenic differentiation of 3T3-L1 and C3H10T1/2 cells, whereas ZFP36L1 overexpression did the opposite. The finding that ZFP36L1 overexpression enhanced osteoblastic and repressed adipogenic differentiation was also corroborated by *ex vivo* experiments. Troglitazone prevented ZFP36L1 from inhibiting adipogenic differentiation, suggesting the significance of PPAR?2 repression in ZFP36L1s inhibitory effect on adipogenic differentiation. ZFP36L1 overexpression repressed the expression of *Ppar?2* mRNA, but not the *PPAR?* promoter activity. Biotin pull-down and electrophoretic mobility-shift assays suggested that ZFP36L1 might interact with endogenous *Ppar?2* mRNA by binding to its 3UTR. The ZFP36L1-containing ribonucleoprotein complexes of ZFP36L1-overexpressing cells contained less *Ppar?2* mRNA than those of control cells. In a luciferase reporter construct, replacement of the SV40 poly(A) fragment by the 3UTR of *Ppar?2* mRNA reduced the expression of luciferase transcripts in ZFP36L1-overexpressing cells. Examination of the kinetic expression of *Ppar?2* mRNA after transcriptional blockage showed that ZFP36L1 might enhance the degradation of the transcripts. Together, these data imply that ZFP36L1 overexpression might repress adipogenesis at least by down-regulating PPAR?2 expression through post-transcriptional mechanisms. Thus, our findings support the notion that decrease of ZFP36L1 expression in bmMSCs with aging might contribute to the aging-related bone loss.

## INTRODUCTION

Aging is associated with degeneration in many organs including bone. Aging bones exhibit reduced bone quality and bone mineral density, which decreases bone strength and renders the elderly prone to have fracture fall. It has been a worldwide task to search for remedies to counteract the aging-related bone deficit. Histological examinations on young and aging bones indicate a difference in the cell composition between the marrows of young and aging bones; aging bones contain more adipocytes but less osteoblasts than young bones [1, 2]. Such a difference could be attributed to the increased adipogenic potential and decreased osteoblastic potential of bone marrow mesenchymal stem cells (bmMSCs), as suggested by the finding that after marrow ablation, bone marrows of aged rats are more adipogenic and less osteoblastogenic than those of young rats [3]. These observations indicate the decrease of osteoblast production as a significant contributor to bone loss in aging, and imply the existence of regulators in promoting adipogenic potential but suppressing osteoblatic potential of bmMSCs as a function of aging. Indeed, a transcription factor known as zinc finger factor 521 has recently been proposed as a potential regulator [4]. Therefore, to elucidate the mechanisms underlying the aging-related bone loss, it would be necessary to identify these regulators.

Zinc finger protein 36, C3H type-like 1 (ZFP36L1) is a member of the tristetraprolin (TTP) family. In rodents, TTP family comprises four members, including TTP, ZFP36L1, ZFP36L2, and ZFP36L3, whereas human TTP family comprises three members (TTP, ZFP36L1, and ZFP36L2) [5, 6]. TTP family members all contain tandem CCCH zinc fingers, which are responsible for RNA-binding. TTP family members can bind to the adenosine and uridine (AU)-rich elements in the untranslated regions (UTRs) of target mRNAs and destabilize the transcripts [7]. Such a capability enables TTP family members to play an important role in the maintenance of normal physiology by decreasing the expression of genes which cause pathological outcomes when excessively expressed. For example, while TTP can bind and destabilize tumor necrosis factor α (TNFα) mRNA to decrease the production of this pro-inflammatory cytokine [8], *TTP*^−/−^ mice produce abundant TNFα, and show symptoms of systemic inflammation [8]. Other than inflammation, TTP family members have also been found to regulate feto-placental development and hematopoiesis, and may also act as tumor suppressors [9–11].

To search for potential regulators of the age-related bone loss, we have recently analyzed the gene expression profiles of human bmMSCs derived from donors of varying ages, and identified a list of potential age-associated genes [12]. We found that *ZFP36L1*, but not *TTP* and *ZFP36L2*, was one of those genes. Interestingly, it was reported that ZFP36L1 expression in osteoblasts was involved in the parathyroid hormone-dependent bone remodeling [13]. These findings suggested a putative role of ZFP36L1 in the maintenance of bone homeostasis and in the etiology of age-related bone loss. However, the role of ZFP36L1 in the regulation of bone formation has not been addressed. ZFP36L1 knockout model was not able to answer this question because it resulted in embryonic lethality. In this regard, studies on the subjects, such as ZFP36L1 expression in bones in relation to age, the impact of ZFP36L1 to osteoblastic and adipogenic differentiation, are expected to provide clues to answer the question. Herein, we investigated the regulatory role of ZFP36L1 in the differentiation of MC3T3-E1 preosteoblasts, 3T3-L1 preadipocytes, and multipotent C3H10T1/2 cells. Our data indicated that ZFP36L1 could act as an enhancer of osteoblastic differentiation but a repressor of adipogenic differentiation, supporting the notion that decreased ZFP36L1 expression in bone marrow stem cells might contribute to aging-related bone loss.

## RESULTS

### ZFP36L1 expression was down-regulated in the femurs and bone marrow mesenchymal stem cells (bmMSCs) of aged rats

**Figure 1:**
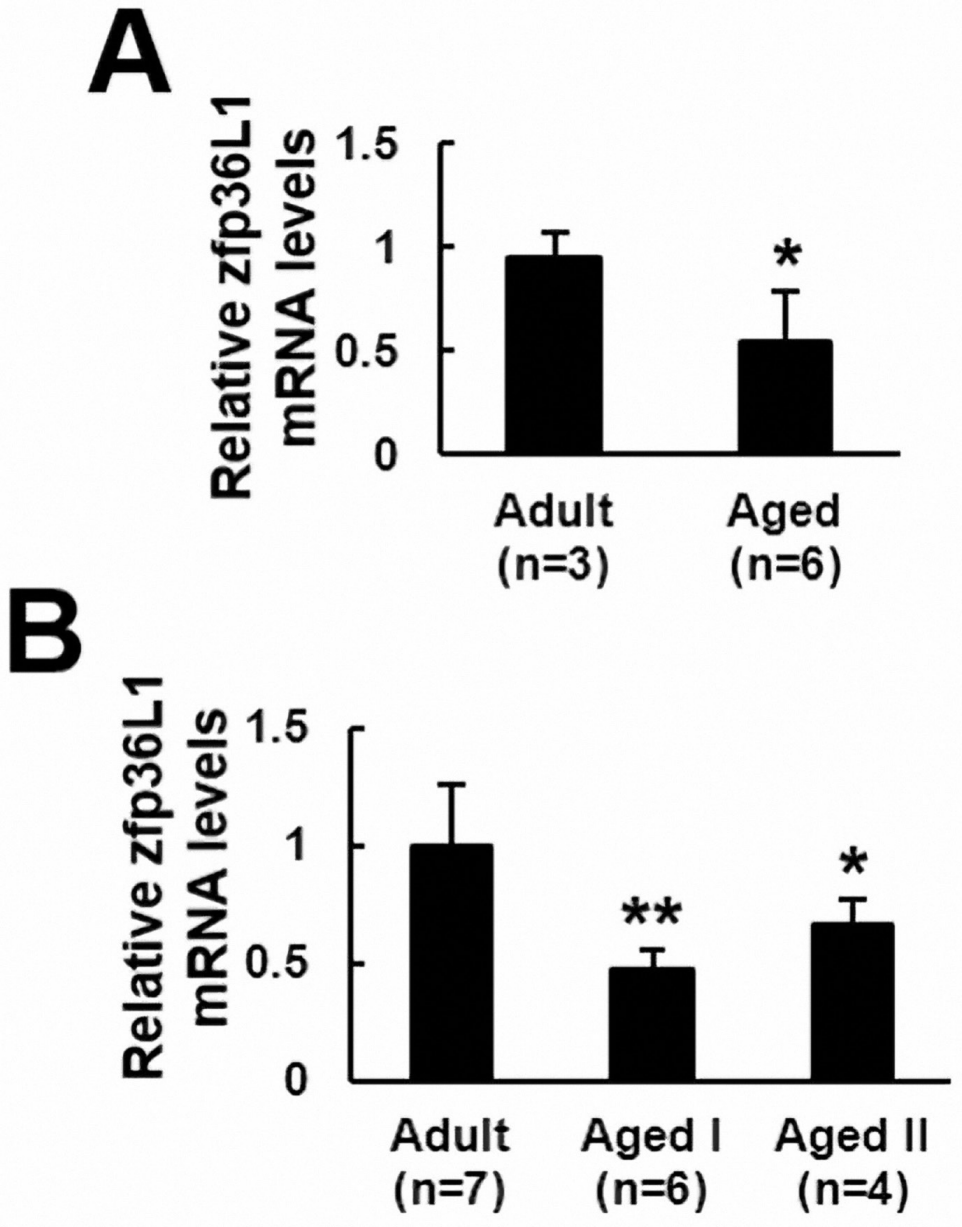
ZFP36l1 expression in the femurs and bmMSCs of adult and aged rats Total RNAs were isolated from the femurs **A**. and bone marrow mesenchymal stem cells **B**. of adult and aged rats, and were subjected to reverse transcription and RT-qPCR analyses for the expression of *Zfp36l1* mRNA. *, *P* < 0.05; **, *P* < 0.001 *versus* adult control.

To examine if ZFP36L1 expression in bones changed with aging, we examined the expression of *Zfp36l1* mRNA in femurs of adult (6-month-old) and aged (18∼22-month-old) rats. Real-time quantitative PCR (RT-qPCR) analyses showed that *Zfp36l1* mRNA level in femurs of aged rats (*n* = 6) was approximately 54.8% of those of adult rats (*n* = 3) (Figure 1A). Next, we pooled bmMSCs isolated from 6-month-old (*n* = 7), 20-month-old (*n* = 6), and 22-month-old (*n* = 4) rats into adult-1, aged-1, and aged-2 groups, respectively, and examined their *Zfp36l1* expression. RT-qPCR analyses showed that *Zfp36l1* mRNA levels in aged-1 and aged-2 groups were approximately 48% and 67%, respectively, of those in adult-1 group (Figure 1B).

### ZFP36L1 enhanced osteoblastic differentiation of MC3T3-E1 preosteoblasts and multipotent C3H10T1/2 cells

**Figure 2:**
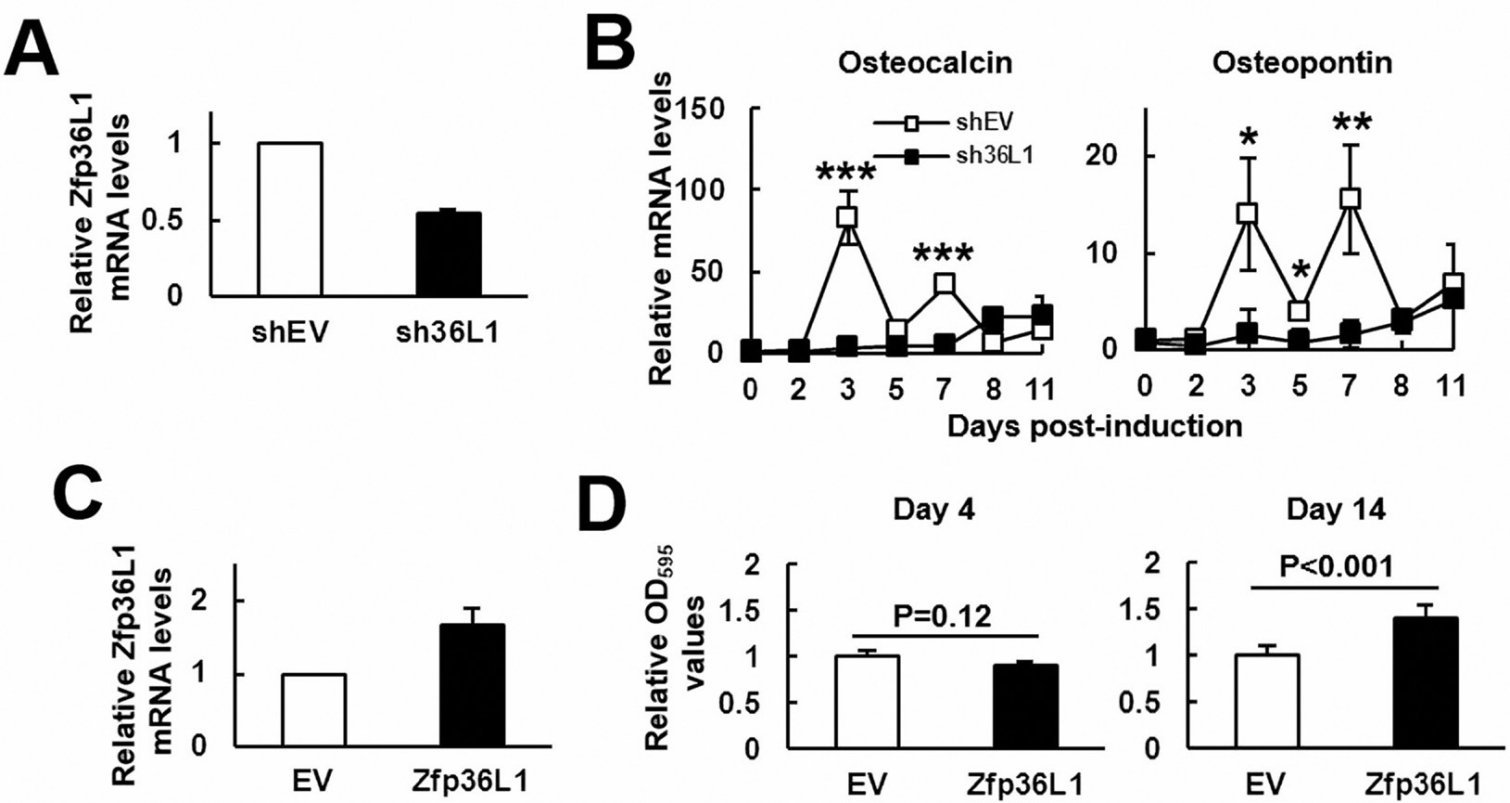
ZFP36L1 regulated osteoblastic differentiation of MC3T3-E1 preosteoblasts **A**. RT-qPCR analyses showed the ZFP36L1 expression of ZFP36L1-knockdown (sh36L1) cells in relation to that of control (shEV) cells. **B**. RT-qPCR analyses. Confluent shEV and sh36L1 cells were induced to undergo osteoblastic differentiation, and the kinetic expression of osteocalcin and osteopontin mRNAs were shown. *, *P* < 0.05; **, *P* < 0.01; ***, *P* < 0.001 *versus* shEV control at day 0. **C**. RT-qPCR analyses showed the ZFP36L1 expression of ZFP36L1-overexpressing (ZFP36L1) cells in relation to that of control (EV) cells. **D**. Quantification of Alizarin Red S stains. Confluent EV and ZFP36L1 cells were induced to undergo osteoblastic differentiation. Cells were stained with Alizarin Red S on days 4 and 14 post-induction. The stains were dissolved and quantitated.

Next, to access the role of ZFP36L1 in osteoblastogenesis, we examined the effect of *Zfp36l1* knockdown on the differentiation of MC3T3-E1 preosteoblasts. We established *Zfp36l1*-knockdown (sh36L1) and control (shEV) cells. *Zfp36l1* mRNA expression of sh36L1 cells was approximately 50% less than that of shEV cells (Figure 2A). We induced cells to undergo osteoblastic differentiation and harvested cells at varying time periods for the measurement of osteocalcin and osteopontin expression. RT-qPCR analyses showed that while osteogenic induction induced dramatic increase of osteocalcin and osteopontin mRNAs around days 3∼7 post-induction, such induction was repressed by *Zfp36l1* knockdown (Figure 2B). Further, we established ZFP36L1-ovexpressing (ZFP36L1) and control (EV) MC3T3-E1 cells. Western blot analyses showed that the level of ZFP36L1 expression in transfected cells was approximately 1.5 fold of that of control cells (Figure 2C). We induced cells to undergo osteoblastic differentiation and examined the calcium precipitation in these cells 4 and 14 days post-induction. As evidenced by the results of Alizarin Red S staining, ZFP36L1-overexpressing cells exhibited stronger differentiation activity than EV cells on day 14 post induction (Figure 2D).

**Figure 3:**
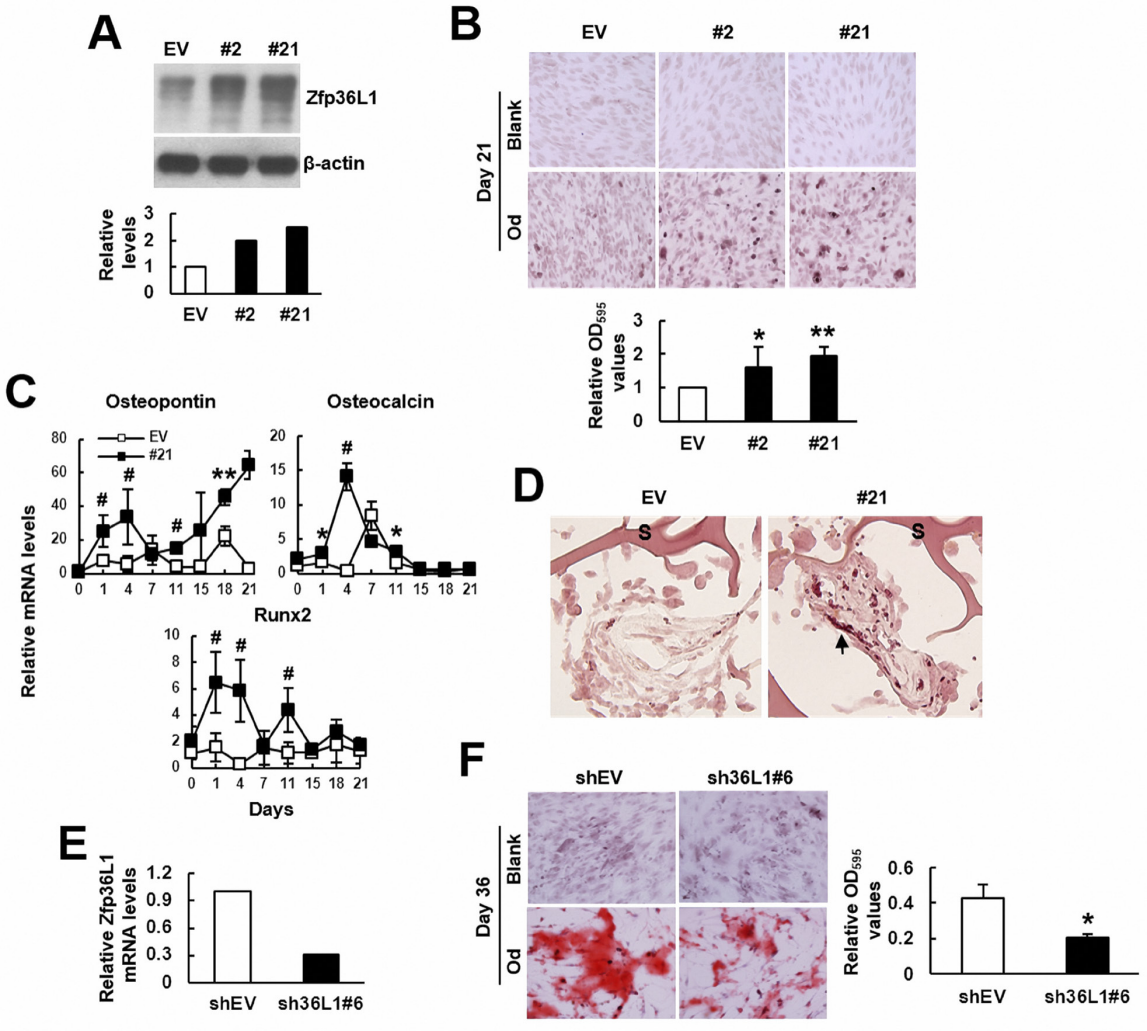
ZFP36L1 regulated osteoblastic differentiation of multipotent C3H10T1/2 cells **A**. Western blot analyses. ZFP36L1 protein levels of EV control, clone 2 (#2), and clone 21 (#21) cells were detected and quantitated. Relative levels were calculated by comparing the signals of #2 and #21 to that of EV cells (to which a value of 1 was assigned). **B**. Osteoblastic induction. EV, #2, and #21 cells were either left untreated (blank) or induced to undergo osteoblastic differentiation (od). Cells were stained with Alizarin Red S 21 days post-induction. Representative photos are shown. The stains were quantitated, and the signals of #2 and #21 were compared to that of EV cells (to which a value of 1 was assigned). *, *P* < 0.05; **, *P* < 0.005 *versus* EV control. **C**. RT-qPCR analyses. The expression kinetics of osteocalcin, osteopontin, and *Runx2* mRNAs in cells after osteoblastic induction were examined, and presented in relative to EV control of day 0 (to which a value of 1 was assigned). *, *P* < 0.05; **, *P* < 0.005; ^#^, *P* < 0.001 *versus* EV control. **D**. *Ex vivo* experiments and histological analyses. EV and #21 cells were seeded into 3 scaffolds (4 × 10^5^ cells/scaffold) separately, and implanted in pairs into the back of 3 nude mice. Implants were retrieved 4 weeks after implantation and prepared for histological analysis. Histological sections were stained with Alizarin Red S. Representative images are shown. Scaffold was indicated by S. Stained cells were indicated by arrowhead. **E**. RT-qPCR analyses showed the ZFP36L1 expression of ZFP36L1-knockdown clone 6 (sh36L1#6) cells in relation to that of control (shEV) cells. **F**. Osteoblastic induction. shEV and sh36L1#6 cells were either left untreated (blank) or induced to undergo osteoblastic differentiation (od). Cells were stained with Alizarin Red S 36 days post-induction. Representative photos are shown. The stains were solubilized and quantitated. *, *P* < 0.05 *versus* shEV control.

Next, to examine if ZFP36L1 also regulated osteoblastic differentiation of C3H10T1/2 cells, we overexpressed ZFP36L1 in C3H10T1/2 cells, and selected two clones (clones 2 and 21) that expressed high levels of *Zfp36l1* mRNA. Western blot analyses showed that ZFP36L1 levels in clones 2 and 21 were approximately 2 and 2.5 fold of those in control cells (Figure 3A). We induced cells to undergo osteoblastic differentiation for 21 days. Alizarin Red S staining suggested that the differentiation activity in clones 2 and 21 was approximately 1.6 and 2 fold, respectively, of that of control cells (Figure 3B). In parallel, we examined the kinetic expression of osteocalcin, osteopontin, and Runx2 mRNAs in clone 21 and control cells by RT-qPCR analyses. In general, data showed that clone 21 expressed more of those mRNAs than control cells (Figure 3C). Moreover, we seeded clone 21 and control cells into scaffolds separately, and implanted subcutaneously into nude mice for 4 weeks. We stained the histological sections of retrieved implants with Alizarin Red S. Unfortunately, cells grew so crowdedly in implants that we were not able to count DAPI-stained cells. However, we observed that ZFP36L1 overexpression seemed to increase the numbers of Alizarin Red S-stained cells (Figure 3D). These results suggested that ZFP36L1 overexpression promoted osteoblastic differentiation of C3H10T1/2 cells. Next, we prepared ZFP36L1-knockdown clone 6 cells whose *Zfp36l1* mRNA levels were approximately 30% of that of the corresponding control cells (Figure 3E). We induced cells to undergo osteoblastic differentiation, and stained cells with Alizarin Red S 36 days post-induction. The results showed that ZFP36L1-knockdown decreased osteoblastic differentiation activity (Figure 3F). Taken together, our data indicated ZFP36L1 as an enhancer of osteoblastic differentiation.

### ZFP36L1 repressed adipogenic differentiation of 3T3-L1 preadipocytes and C3H10T1/2 cells

**Figure 4:**
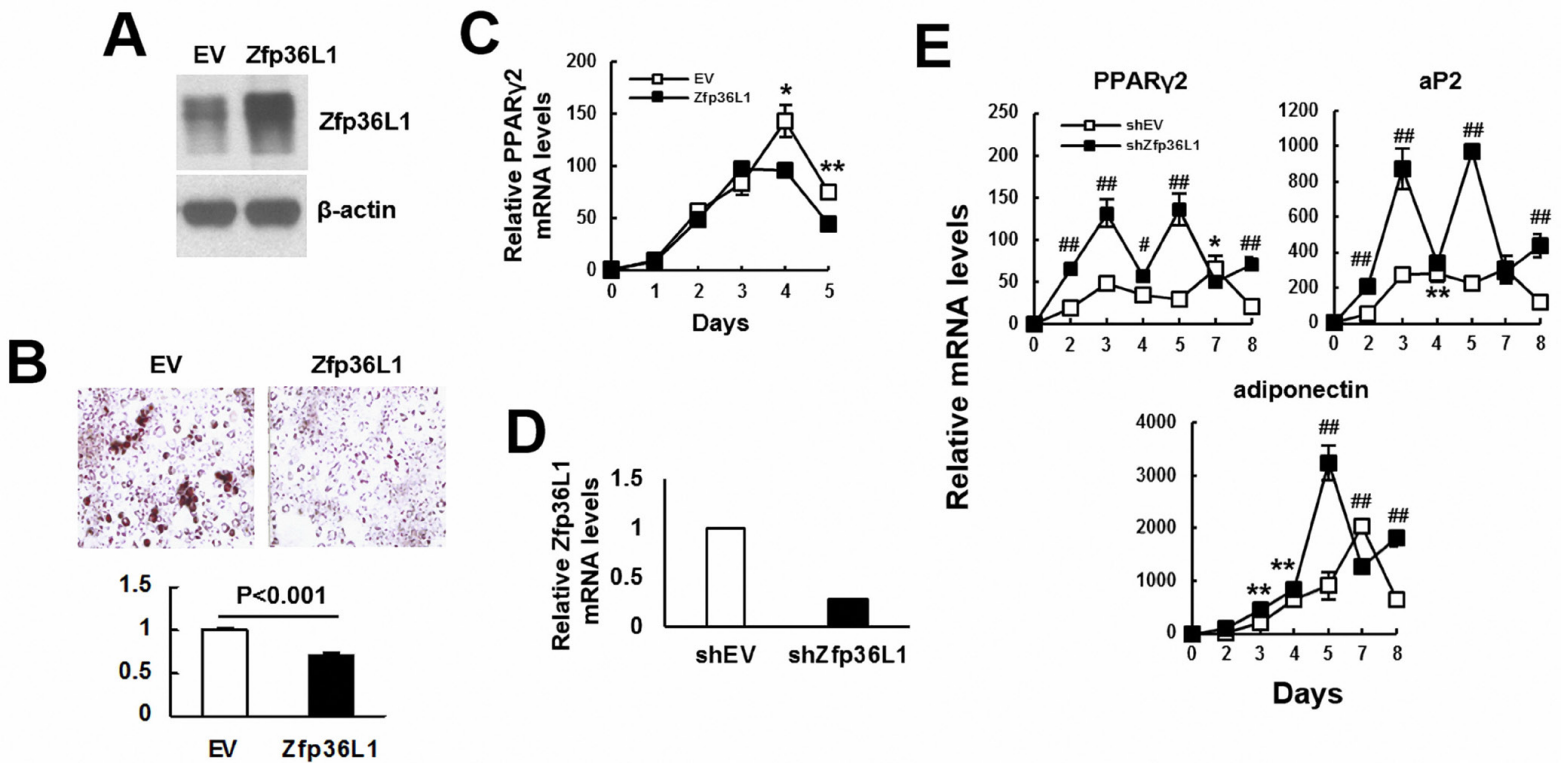
ZFP36L1 regulated adipogenic differentiation of 3T3-L1 preadipocytes **A**. Western blot analyses. ZFP36L1 protein levels of EV control and ZFP36L1-overexpressing (ZFP36L1) 3T3-L1 cells were shown. **B**. Adipogenic induction. EV and ZFP36L1 cells were induced to undergo adipogenic differentiation. Cells were stained with Oil Red O 5 days post-induction. Representative photos are shown. The stains were quantitated, and the signal of ZFP36L1 cell was compared to that of EV cell (to which a value of 1 was assigned). **C**. RT-qPCR analyses. The expression kinetics of *Pparγ2* mRNA in cells after adipogenic induction were examined, and presented in relative to EV control of day 0 (to which a value of 1 was assigned). *, *P* < 0.05; **, *P* < 0.01 *versus* EV control. **D**. RT-qPCR analyses. ZFP36L1 expression of ZFP36L1-knockdown (shZFP36L1) cells in relation to that of control (shEV) cells was shown. **E**. RT-qPCR analyses. shEV and shZFP36L1 cells were induced to undergo adipogenic differentiation. The expression kinetics of *Pparγ2*, *aP2*, and adiponectin mRNAs in cells after adipogenic induction were examined, and presented in relative to EV control of day 0 (to which a value of 1 was assigned). *, *P* < 0.05; **, *P* < 0.01; ^#^, *P* < 0.001; ^##^, *P* < 0.0001 *versus* EV control.

We induced C3H10T1/2 and 3T3-L1 cells to undergo adipogenic differentiation, and examined the kinetic expression of *Zfp36l1* mRNA levels. The results showed that the expression of *Zfp36l1* mRNA decreased with the advance of differentiation in these cells (Supplemental Figure S1). To assess the role of ZFP36L1 in adipogenic differentiation, we established ZFP36L1-ovexpressing and control 3T3-L1 cells. Western blot analyses showed that the ZFP36L1 level of ZFP36L1-ovexpressing cells was approximately 2.2 fold of that of control cells (Figure 4A). We induced cells to undergo adipogenic differentiation for various days, and examined lipid droplet formation as well as *Pparγ2* mRNA expression. As evidenced by Oil Red O staining performed 5 days post induction, ZFP36L1-ovexpressing cells showed approximately 28% less lipid droplet formation than control cells (Figure 4B). RT-qPCR analyses showed that adipogenic induction increased *Pparγ2* expression in both groups of cells; however, ZFP36L1 overexpression significantly repressed *Pparγ2* expression 4 days post induction (Figure 4C). We also prepared ZFP36L1-knockdown cells whose *Zfp36l1* mRNA levels were approximately 72% less than control 3T3-L1 cells (Figure 4D), and induced cells to undergo adipogenic differentiation for various days. RT-qPCR analyses showed that ZFP36L1 knockdown potentiated the mRNA expression of PPARγ2, aP2, and adiponectin (Figure 4E).

**Figure 5:**
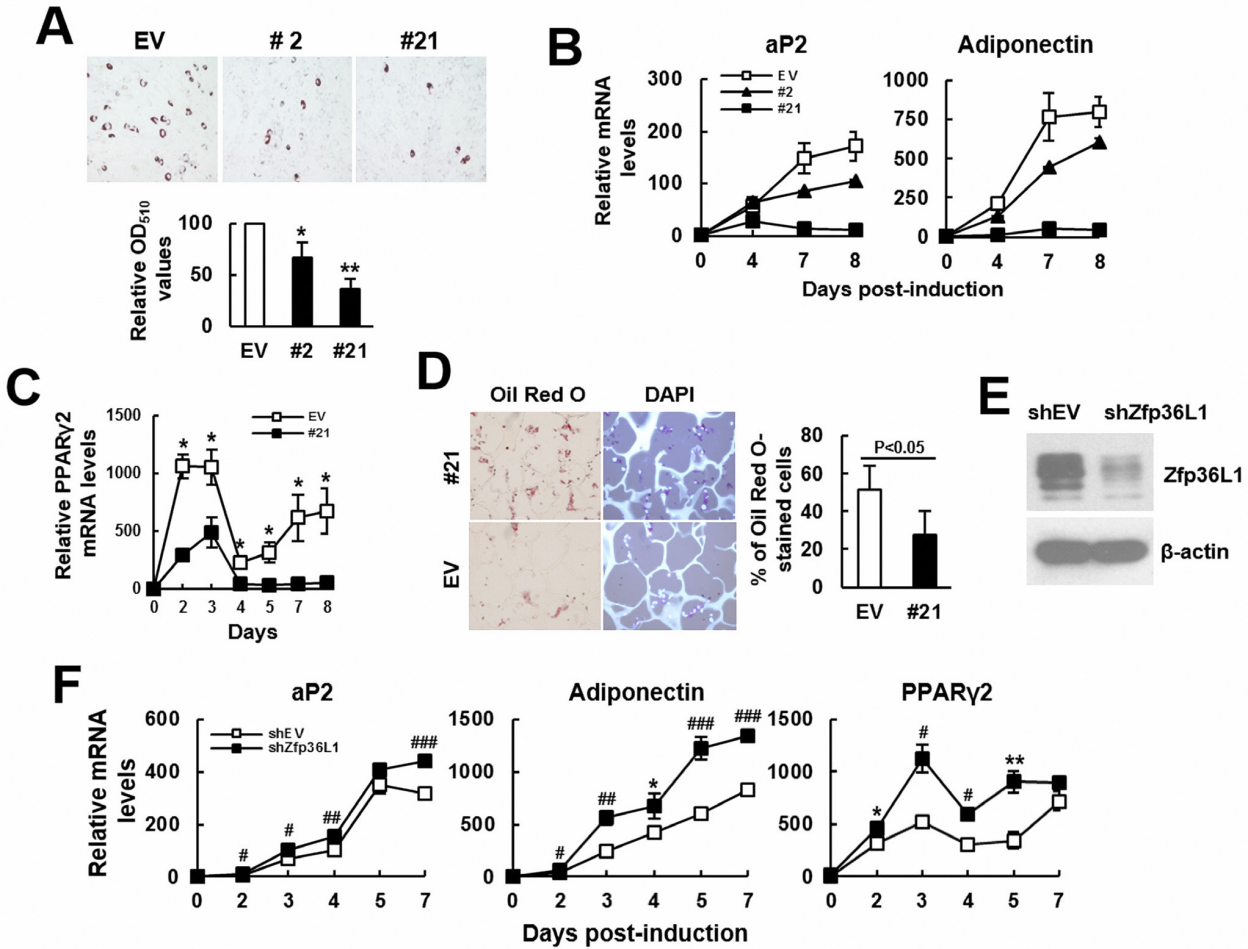
ZFP36L1 regulated adipogenic differentiation of C3H10T1/2 cells **A**. Adipogenic differentiation. C3H10T1/2 control (EV), clone 2 (#2), and clone 21(#21) cells were induced to undergo adipogenic differentiation. Cells were stained with Oil Red O 8 days post-induction. Representative photos are shown. The stains were quantitated, and the signals of #2 and #21 were compared to that of EV cells (to which a value of 100 was assigned). *, *P* < 0.05; **, *P* < 0.005 *versus* EV control. **B**. RT-qPCR analyses. The expression kinetics of *aP2* and adiponectin mRNAs in cells after adipogenic induction were examined, and presented in relative to EV control of day 0 (to which a value of 1 was assigned). **C**. RT-qPCR analyses. The expression kinetics of *Pparγ2* mRNA in EV and #21 cells after adipogenic induction were shown. *, *P* < 0.05 *versus* EV control. **D**. *Ex vivo* experiments and histological analyses. EV and #21 cells were seeded into 3 scaffolds (4 × 10^5^ cells/scaffold) separately, and implanted in pairs into the back of 3 nude mice. Implants were retrieved 2 weeks after implantation and prepared for histological analysis. Histological sections were stained with Oil Red O and DAPI. Representative images are shown. The ratio of Oil Red O-stained cells were calculated. **E**. Western blot analyses. ZFP36L1 expression of ZFP36L1-knockdown (shZFP36L1) and control (shEV) C3H10T1/2 cells were shown. **F**. RT-qPCR analyses. shEV and shZFP36L1 cells were induced to undergo adipogenic differentiation. The expression kinetics of *aP2*, adiponectin, and *Pparγ2* mRNAs were shown. *, *P* < 0.05; **. *P* < 0.01; ^#^, *P* < 0.005; ^##^, *P* < 0.001; ^###^, *P* < 0.0001 *versus* corresponding EV control.

Next, we examined the impact of ZFP36L1 overexpression to the adipogenic differentiation of the ZFP36L1-overexpressing C3H10T1/2 clones 2 and 21. We induced cells to undergo adipogenic differentiation for various days. Oil Red O staining performed 8 days post-induction showed that the accumulated lipid droplets in clones 2 and 21 was approximately 67% and 36%, respectively, of that of control cells (Figure 5A). In parallel, RT-qPCR analyses showed that adipogenic induction increased the mRNA levels of aP2 and adiponectin, whereas ZFP36L1 overexpression repressed expression of these genes in a dose-dependent manner (Figure 5B). Moreover, comparison of the kinetic expression of *Pparγ2* mRNA in clone 21 and control cells also showed decreased PPARγ2 expression in clone 21 cells (Figure 5C). Subsequently, we seeded clone 21 and control cells into scaffolds separately, and implanted subcutaneously into nude mice for 2 weeks. We stained the histological sections of retrieved implants with DAPI and Oil Red O, and to estimate the percentage of Oil Red O-stained cells in the histological sections. Our data showed that approximately 51% and 27% of DAPI-stained cells in control and clone 21 groups, respectively, accumulated lipid droplets (Figure 5D), indicating that ZFP36L1 overexpression inhibited adipogenic differentiation of C3H10T1/2 cells. On the other hand, we prepared ZFP36L1-knockdown cells whose ZFP36L1 levels were approximately 72% less than control cells (Figure 5E), and induced cells to undergo adipogenic differentiation for various days. RT-qPCR analyses showed that Zfp36L1 knockdown increased the mRNA levels of aP2, adiponectin and PPARγ2 (Figure 5F).

**Figure 6:**
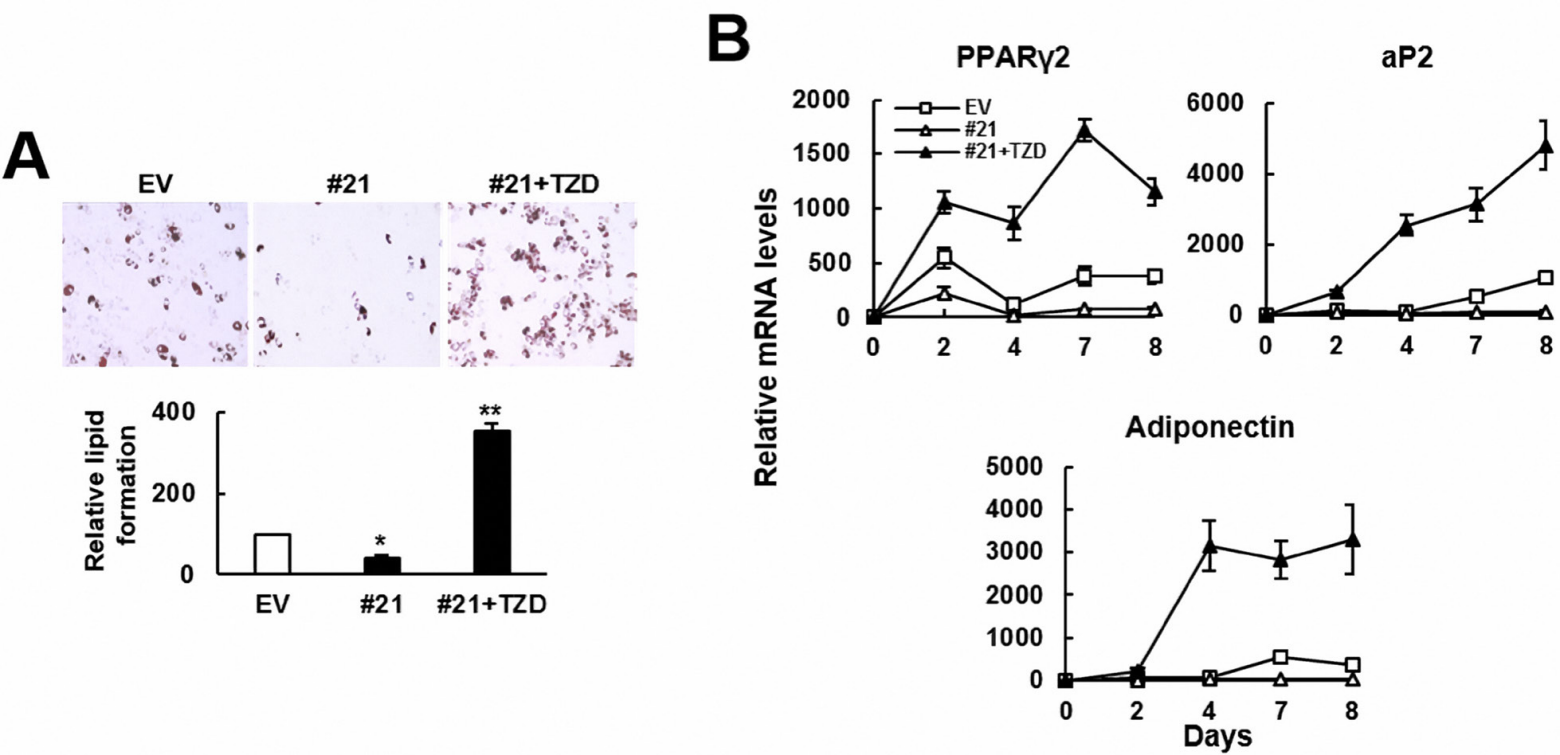
TZD prevented ZFP36L1 from inhibiting adipogenic differentiation of C3H10T1/2 cells **A**. Adipogenic differentiation. Control (EV) and ZFP36L1-overexpressing C3H10T1/2 #21 cells (#21) were induced to undergo adipogenic differentiation, and #21 cells were either left untreated or concomitantly treated with troglitazone (TZD, 1 μM). Cells were stained with Oil Red O 8 days post-induction. Representative photos are shown. The stains were quantitated, and the signals of #21 cells were compared to that of EV cells (to which a value of 100 was assigned). *, *P* < 0.05; **, *P* < 0.00005 *versus* EV control. **B**. RT-qPCR analyses. The expression kinetics of *Pparγ2*, *aP2*, and adiponectin mRNAs in cells after adipogenic induction were shown.

Next, we examined if troglitazone (TZD), a PPARγ2 agonist, prevented ZFP36L1 from inhibiting adipogenic differentiation. Our data showed that while ZFP36L1 overexpression inhibited lipid droplets formation and the expression of PPARγ2, aP2, and adiponectin, TZD treatment restored the adipogenic differentiation (Figure 6). These evidences suggested the significance of PPARγ2 repression in ZFP36L1′s inhibitory effect on adipogenic differentiation.

### ZFP36L1 regulated PPARγ2 expression by post-transcriptional mechanism(s)

**Figure 7:**
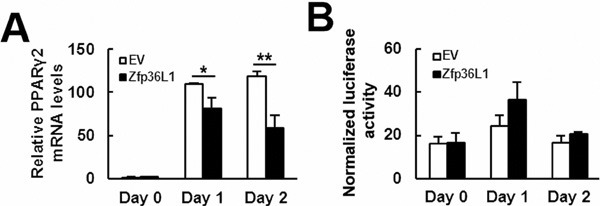
ZFP36L1 decreased PPARγ2 expression but not Pparγ promoter activity ZFP36L1-overexpressing (ZFP36L1) and control (EV) C3H10T1/2 cells were transfected with a luciferase reporter driven by *Pparγ* promoter and a Renilla control reporter, cells were then induced to undergo adipogenic differentiation, and were harvested at the times indicated. **A**. RT-qPCR analyses. The expression of *Pparγ2* mRNA was examined. Data represent the mean ± S.D. from three experiments. *, *P* < 0.05; ^*^, *P* < 0.005 *versus* control. **B**. Luciferase assays. Cells harvested after adipogenic induction were analyzed for luciferase and Renilla activities. The normalized luciferase signals of EV and ZFP36L1 cells were shown.

**Figure 8:**
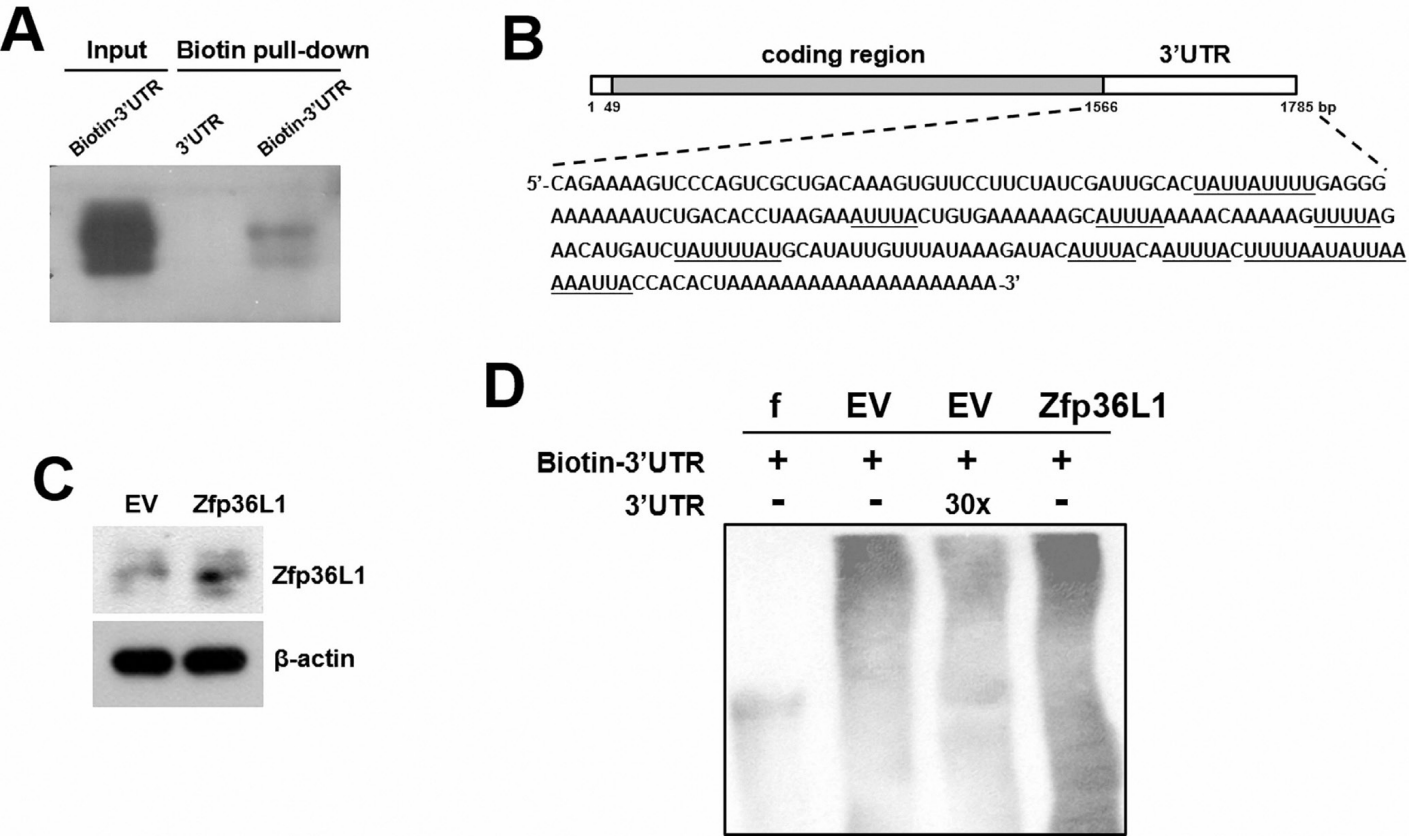
Interactions of ZFP36L1 and Pparγ2 transcripts **A**. Biotin pull-down assays. C3H10T1/2 cells were lysed, and 150 μg of cell lysates were incubated with either biotin-labeled (biotin-3′UTR) or unlabeled (3′UTR) transcripts derived from the *Pparγ2* mRNA, and were subjected to pull-down assays followed by Western blot analyses to detect ZFP36L1 expression. Forty micrograms of whole-cell lysate was used as input. **B**. Schematic representation of the full-length *Pparγ2* mRNA. The sequence of the 3′UTR is shown, and in which the putative AU-rich fragments are underlined. **C**. Western blot analyses. The ZFP36L1 levels of EV control cells and cells transiently overexpressing ZFP36L1 (ZFP36L1) were examined. **D**. RNA EMSA assays. Ten-micrograms of cytoplasmic fractions prepared from control (EV) and ZFP36L1-overexpressing (ZFP36L1) cells were reacted with biotin-labeled 3′UTR of *Pparγ2* mRNA. A 30-molar excess of unlabeled 3′UTR probes was added for competition experiments. f, biotin-labeled transcripts without incubation with cell lysate.

**Figure 9:**
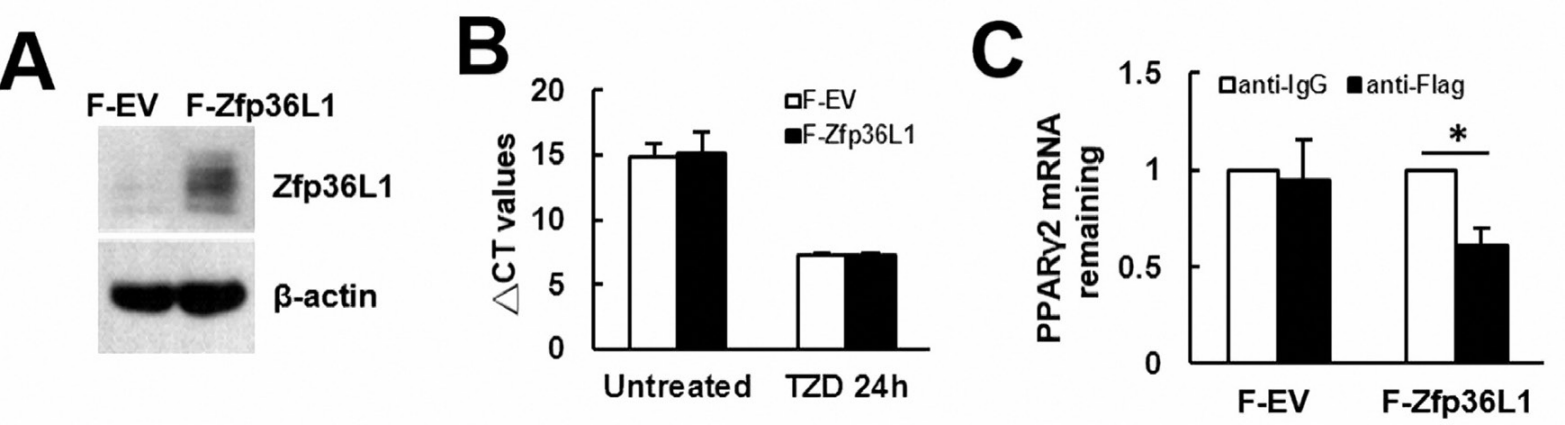
Examination of the binding of ZFP36L1-containing protein complexes on *Pparγ2* mRNA **A**. Western blot analyses. The ZFP36L1 levels of C3H10T1/2 cells overexpressing Flag-tagged ZFP36L1 (F-ZFP36L1) and control cells (F-EV) were shown. **B**. RT-qPCR analyses. F-EV and F-ZFP36L1 cells were induced to undergo adipogenic differentiation with or without concomitant treatment of troglitazone (TZD, 1 μM) for 24h. The expression of *Pparγ2* mRNA was examined. **C**. Ribonucleoprotein immunoprecipitation and RT-qPCR analyses. One milligram of lysates prepared from F-EV or F-ZFP36L1 cells (1 × 10^7^) were incubated with protein A beads precoated with 15 μg of either anti-Flag or anti-IgG antibody to precipitate ribonucleoprotein complexes and to extract RNAs from the complexes as described in Materials and Methods. RNAs were used in subsequent RT-qPCR assays for *Pparγ2* and β-actin mRNAs. *Pparγ2* signals were normalized to β-actin signals. The normalized *Pparγ2* signals obtained from the ribonucleoprotein complexes pulled down by anti-Flag antibody were compared with those pulled down by anti-IgG antibody (to which a value of 1 was assigned). Data represent the mean ± S.D. from three analyses. *, *P* < 0.05.

**Figure 10:**
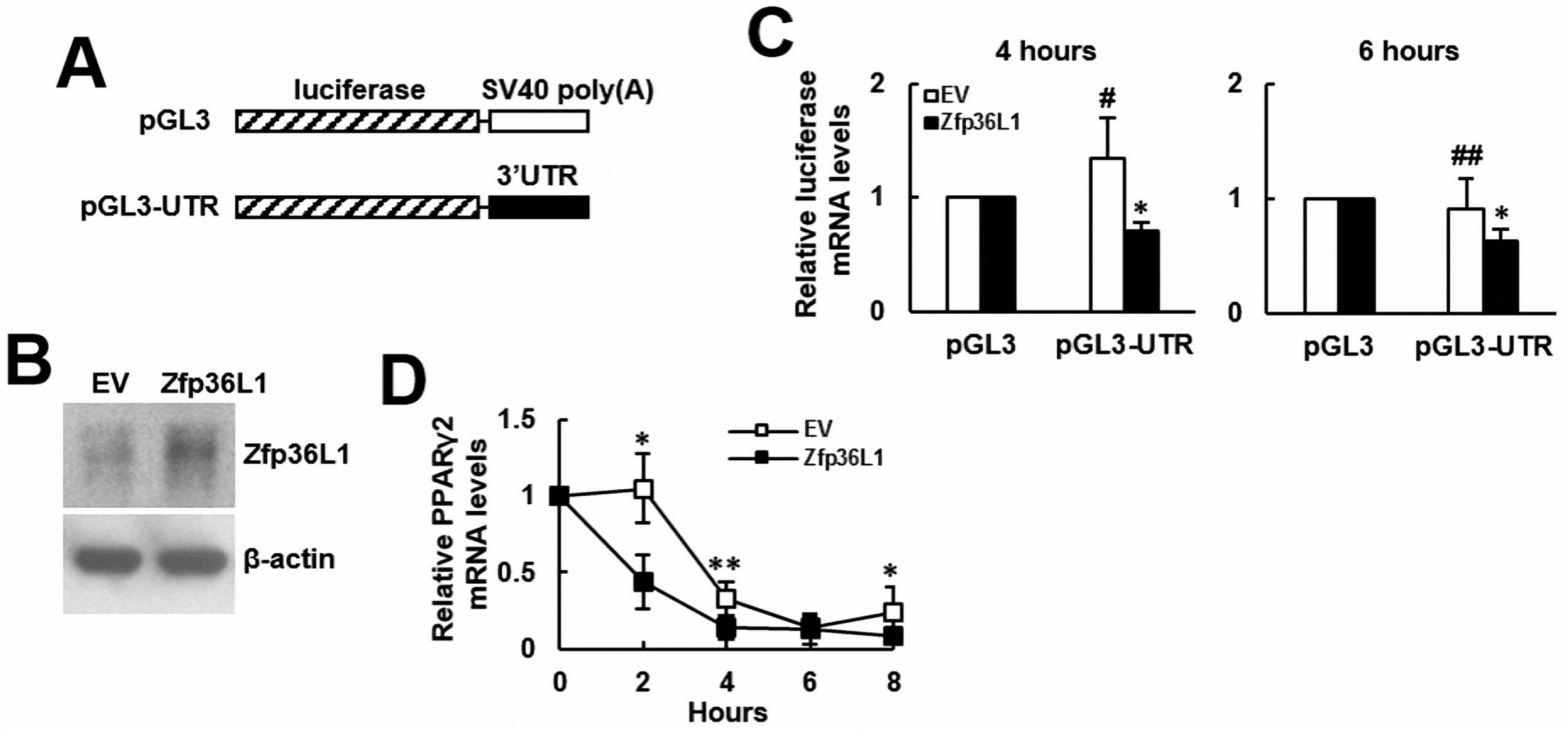
Binding of ZFP36L1-containing protein complexes on the 3′UTR of Pparγ2 mRNA down-regulated gene expression **A**. Schematic representation of the pGL3 control and pGL3-UTR constructs. **B**. Western blot analyses. The ZFP36L1 levels in C3H-EV (EV) and C3H-ZFP36L1 (ZFP36L1) cells were shown. **C**. RT-qPCR analyses. ZFP36L1-overexpressing (ZFP36L1) and control (EV) C3H10T1/2 cells were transfected with 2 mg of either pGL3-promoter (pGL3) or pGL3-UTR construct along with 0.1 mg of a Renilla luciferase reporter as a transfection control. Cells were harvested 4 and 6 h post transfection, and the levels of the luciferase transcripts were examined. Luciferase levels were normalized to the levels of Renilla and β-actin. The normalized luciferase signals of cells receiving pGL3-UTR were compared to the normalized luciferase signals of cells receiving pGL3 (to which a value of 1 was assigned). Data represent the mean ± S.D. from three analyses. *, *P* < 0.01; ^#^, *P* = 0.479; ^##^, *P* = 0.239 *versus* control. **D**. RT-qPCR analyses. ZFP36L1-overexpressing (ZFP36L1) and control (EV) C3H10T1/2 cells were induced to undergo adipogenic differentiation with concomitant treatment of troglitazone (1 μM) for 24h. Cells were then treated with Actinomycin D (1 μg/ml) and were harvested 0, 2, 4, 6, and 8 h post treatment. The relative expression levels of *Pparγ2* mRNA were calculated in relation to the level at 0 h (to which a value of 1 was assigned). Data represent the mean ± S.D. from three experiments. *, *P* < 0.05; ^*^, *P* < 0.01 *versus* corresponding EV control.

To elucidate how ZFP36L1 regulated lineage differentiation, we focused on how ZFP36L1 regulated PPARγ2 expression. We overexpressed ZFP36L1 followed by an empty luciferase reporter or a reporter driven by *Pparγ* promoter in parental cells transiently, and induced cells to undergo adipogenic differentiation, and harvested cells 0, 1, and 2 days post-induction. RT-qPCR analyses showed that the induction of PPARγ2 expression as shown in the control cells was repressed in cells receiving ZFP36L1 (Figure 7A). Parallel luciferase assays showed that the *Pparγ* promoter activity in cells receiving ZFP36L1 was no less than that of control cells, indicating that ZFP36L1 did not inhibit *Pparγ* promoter activity (Figure 7B). Taken together, these data indicated that ZFP36L1 might inhibit PPARγ2 expression at the post-transcriptional level. So, we performed biotin pull-down assays using unlabeled and biotin-labeled 3′UTR of *Pparγ2* mRNA to examine the interaction between ZFP36L1 and *Pparγ2* mRNA. Western blot analyses on the complexes pulled down by streptavidin-coated beads showed that ZFP36L1 was detected in the complexes reacting with biotin-labeled transcript but not in those reacting with unlabeled transcript, which indicated the binding specificity of the streptavidin-coated beads, and suggested the binding of endogenous ZFP36L1 to *Pparγ2* mRNA (Figure 8A). The 3′UTR of *Pparγ2* mRNA contains several AU-rich fragments (Figure 8B). To further address the binding between ZFP36L1 and *Pparγ2* mRNA, we performed electrophoretic mobility-shift assays. We prepared the cytoplasmic components of the cell lysates prepared from the control and the cells transiently overexpressed ZFP36L1 (Figure 8C) and incubated these components with biotin-labeled 3′UTR of *Pparγ2* mRNA. As shown in Figure 8D, cytoplasmic lysates of the control cells bound to the biotin-labeled RNA probes. A 30-fold molar excess of unlabeled RNA probes decreased the binding to the biotin-labeled RNA probes, which resulted in the appearance of unbound free biotin-labeled RNA probes. In addition, cytoplasmic lysates of the ZFP36L1-overexpressing cells exhibited stronger RNA-binding activity than the lysates of control cells (Figure 8D). Our data suggested the binding of ZFP36L1 to the 3′UTR of *Pparγ2* mRNA. Subsequently, we prepared Flag-tagged ZFP36L1-overexpressing (F-ZFP36L1) and control (F-EV) C3H10T1/2 cells (Figure 9A), and induced these cells to undergo adipogenic differentiation for 24 h at the presence of troglitazone. We harvested cells 24 h post-induction. RT-qPCR assays revealed that troglitazone induced equal amounts of *Pparγ2* mRNA in F-EV and F-ZFP36L1 cells (Figure 9B). Then, we performed immunoprecipitation experiments using anti-Flag or control antibody to pull down the ribonucleoprotein complexes in equal amounts of lysates prepared from these cells. RNAs were extracted from these immunoprecipitates, and subjected to RT-qPCR analyses for *Pparγ2* and β-actin mRNAs. The results showed that in F-EV cells, there was no significant difference in the amounts of *Pparγ2* mRNA between the ribonucleoprotein complexes pulled down by anti-Flag and control antibody, respectively (Figure 9C). However, in F-ZFP36L1 cells, the levels of *Pparγ2* mRNA in the ribonucleoprotein complexes pulled down by anti-Flag antibody was 40% less than that in the ribonucleoprotein complexes pulled down by control antibody (Figure 9C). A possible explanation for this result is that the F-ZFP36L1-containing protein complexes were able to enhance the degradation of *Pparγ2* mRNA. Accordingly, we set out to examine the function of the 3′UTR of *Pparγ2* mRNA in regulating gene expression in the background of ZFP36L1 overexpression. We replaced the SV40 poly(A) fragment which located 3′ to the luciferase cDNA in pGL3-promoter vector by the 3′UTR of *Pparγ2* mRNA to generate pGL3-UTR reporter (Figure 10A). We established ZFP36L1-overexpressing C3H-ZFP36L1 and control C3H-EV cells (Figure 10B), transfected these cells with pGL3-UTR or control luciferase reporter, and then quantitated the levels of luciferase transcripts by RT-qPCR analyses. As examined 4 and 6 h post transfection, and compared with the luciferase transcript levels of C3H-EV cells receiving control pGL3 reporter, the levels of luciferase transcript in C3H-EV cells receiving pGL3-UTR reporter were not significantly changed (Figure 10C). On the other hand, compared with the luciferase transcript levels of C3H-ZFP36L1 cells receiving control pGL3 reporter, the levels of luciferase transcript in C3H-ZFP36L1 cells receiving pGL3-UTR reporter were approximately 29% and 37% lower, as examined 4 and 6 h post transfection, respectively (Figure 10C). We also induced C3H-EV and C3H-ZFP36L1 cells to undergo adipogenic differentiation at the presence of troglitazone which was removed 24 h post induction. We treated cells with Actinomycin D for 0, 2, 4, 6, and 8 h, and examined the *Pparγ2* mRNA levels. As shown in Figure 10D, *Pparγ2* mRNA levels in C3H-EV cells remained intact 2 h after Aitinomycin D treatment, and decreased ∼67%, ∼85% and ∼76% at 4, 6, and 8 h after treatment, respectively. On the other hand, *Pparγ2* mRNA levels in C3H-ZFP36L1 cells decreased ∼56%, ∼86%, ∼87%, and ∼92% at 2, 4, 6, and 8 h after Actinomycin D treatment. Taken together, our data suggested that ZFP36L1 might down-regulate the expression of *Pparγ2* mRNA by binding to the 3′UTR of this mRNA.

## DISCUSSION

As ZFP36L1 is a RNA-binding protein, it is expected to be able to modulate the levels of its target transcripts and therefore participate in a wide spectrum of physiological activities. While ZFP36L1 and the other TTP members have been found to harbor anti-inflammatory and anti-cancer capability, we have explored the potential involvement of ZFP36L1 in the regulation of aging-related bone loss.

It is well documented that aging bones contain less bone mass and osteoblasts than adult bones, which can be attributed to decreased bone formation in aging bones. Bone formation is carried out by osteoblasts which are differentiated from osteoprogenitors and bmMSCs. So, if ZFP36L1 played a role in the aging-related bone loss, it would be reasonable to assume that ZFP36L1 might express differentially in adult and aged bmMSCs, and that ZFP36L1 might regulate osteoblastic and adipogenic differentiation. Notably, our data showed that expression of *Zfp36l1* mRNA was down-regulated in the femurs and bmMSCs of aged rats compared with those noted in adult rats (Figure 1). Moreover, by examining the impact of ZFP36L1 knockdown and overexpression on osteoblastic differentiation of MC3T3-E1 and C3H10T1/2 cells *in vitro*; and on the osteoblastogenic potential of C3H10T1/2 cells *ex vivo* (Figures 2 and 3), we showed that ZFP36L1 could act as an enhancer of osteoblastic differentiation. On the other hand, examinations of ZFP36L1 knockdown and overexpression on adipogenic differentiation of 3T3-L1 and C3H10T1/2 cells *in vitro*; and on the adipogenic potential of C3H10T1/2 cells *ex vivo* (Figures 4 and 5), we showed that ZFP36L1 could act as a repressor of adipogenic differentiation. Taken together, our findings support the notion that ZFP36L1 might participate in the maintenance of bone homeostasis, and that down-regulation of ZFP36L1 expression in bmMSCs might be a part of the mechanisms underlying the aging-related bone loss.

Our studies also shed light on the mechanisms by which ZFP36L1 regulates lineage differentiation. Runx2 is the master transcriptional regulator of osteoblastic differentiation; it can promote osteoblastic differentiation by recruiting co-activators [14]. Our data showed that ZFP36L1 overexpression increased *Runx2* mRNA expression (Figures 3C). Given that ZFP36L1 is a RNA-binding protein, and that binding of ZFP36L1 enhances the degradation of its target transcripts, ZFP36L1 is unlikely to increase Runx2 expression by stabilizing *Runx2* mRNA. How ZFP36L1 induces Runx2 expression, and more importantly, how critical the Runx2 induction is in the ZFP36L1-induced commitment to osteoblastic lineage are currently unclear. In comparison, however, it has been shown that PPARγ2 is a more dominant regulator of osteoblastic differentiation. PPARγ2 overexpression repress Runx2 expression and converts osteoblastic cells to adipocytes [15]. Partially knockout of *Pparγ* enhances bone formation in transgenic mice through increased osteoblastogenesis [16]. With these evidences in mind, our data, which showed that ZFP36L1 overexpression decreased *Pparγ2* mRNA expression, whereas ZFP36L1 knockdown increased *Pparγ2* mRNA expression and decreased osteoblastic differentiation, and that TZD prevented ZFP36L1 from inhibiting adipogenic differentiation, support the speculation that down-regulation of PPARγ2 expression could be a critical step for ZFP36L1 to repress adipogenic lineage but to promote osteoblastic lineage. Moreover, by showing that (i) ZFP36L1 overexpression decreased PPARγ2 expression without concomitantly decreasing *Pparγ* promoter activity (Figure 7), (ii) endogenous ZFP36L1 interacted with *Pparγ2* mRNA and bound its 3′UTR (Figure 8), and (iii) ZFP36L1 overexpression inhibited the expression of a luciferase transcript containing the 3′UTR of *Pparγ2* mRNA, and enhanced the degradation of *Pparγ2* mRNA (Figure 10), our data suggest that ZFP36L1 regulates adipogenic differentiation at post-transcriptional level, and that ZFP36L1 might interact with the 3′UTR of *Pparγ2* mRNA, presumably by binding to the AU-rich element(s), and mediate its degradation.

On the other hand, it is noteworthy that Runx2 can also repress osteoblastic differentiation once recruiting co-repressors. For examples, HDACs such as HDAC3 and HDAC7 bind to Runx2 and prevent it from transactivating the expression of osteocalcin and osteopontin, respectively [17, 18]. Interestingly, we found that ZFP36L1 overexpression in C3H10T1/2 cells decreased the expression of *Hdac3* and *Hdac7* mRNAs during osteoblastic differentiation (Supplemental Figure S2), which supports, in part, the possibility that ZFP36L1 might be able to decrease the expression of HDACs to release Runx2′s transcriptional activity from the inhibitory HDACs’ effect.

While our studies focused on the role of ZFP36L1 in aging-related bone loss, the finding that ZFP36L1 was able to regulate adipogenesis raises a question as to whether this molecule may also play a potential role in obesity. Several microRNAs have been found to enhance or repress adipogenesis of preadipocytes, MSCs, obese adipose tissues, and obese adipocytes depending on their targets [20, and the references therein]. Recently, Di Bernardo et al. reported that the sera of overweight individuals was able to promote the adipogenic differentiation of bmMSCs while partially repress osteogenesis [21]. Therefore, it will be of interest to examine if ZFP36L1 is involved in the mechanisms by which microRNAs and the circulating factors regulate adipogenesis and obesity. Besides, ZFP36L1 has also been shown to degrade the transcripts of numerous senescence-associated secretory phenotype (SASP) components which otherwise enhance senescence [22], which indicates a regulatory role of Zfp36L1 in senescence. Accordingly, we overexpressed Zfp36L1 in the bmMSCs derived from aged rats (Supplemental Figure S3A), and stained the cells for the senescence-associated β-galactosidase. The results showed that Zfp36L1 overexpression decreased the number of stained cells (Supplemental Figure S3B). We also examined the expression of Cdkn1a and Cdkn2a (cell cycle inhibitors), Rb1, and Rb2. Our data showed that ZFP36L1 overexpression decreased the expression of *Cdkn1a*, *Cdkn2a*, and *Rb2* mRNAs, but increased the expression of *Rb1* mRNAs (Supplemental Figure S3C). It has been reported by Galderisi et al. that Rb1 expression in MSCs decreased during *in vitro* cultivation, and that Rb2/P130 may play a role in triggering the senescence process in MSCs [23]. Therefore, our data support the notion that down-regulation of Zfp36L1 may be a contributor to the development of senescence phenotype in bmMSCs as well.

In summary, we have shown the aging-associated expression pattern of ZFP36L1, and conducted *in vitro* and *ex vivo* experiments to address ZFP36L1′s candidacy as an enhancer of osteoblastic differentiation but a repressor of adipogenic differentiation of multipotent cells. These findings, together with the finding that ZFP36L1 was able to bind to the 3′UTR and target *Pparγ2* mRNA for down-regulation, link this RNA-binding protein to an aging phenotype (bone loss), and support the notion that decreased ZFP36L1 expression in bmMSCs might contribute to aging-related bone loss. Since that *Zfp36l1* knockout is embryonically lethal, it would be necessary to establish mouse models allowing for conditional knockout of ZFP36L1 in bones to verify the role of ZFP36L1 in regulating bone formation in aging. Beyond this, our data provide an experimental basis to further delineate the functional significance of ZFP36L1 in aging.

## MATERIALS AND METHODS

### Plasmids

Full-length mouse *Zfp36l1* cDNA was cloned into pcDNA3.1(-) vector (Invitrogen, CA, USA) to generate pcDNA3.1-ZFP36L1 for ZFP36L1 overexpression in C3H10T1/2 cells. The cDNA was also cloned into pLAS2w.Pneo vector to generate pLAS2w-ZFP36L1 for Lentivirus preparation. pLAS2w.Pneo and pshRNAZFP36L1 (a plasmid harboring shRNA targeting *Zfp36l1* mRNA for degradation) were purchased from the National RNAi Core Facility at Academia Sinica, Taiwan. A cDNA fragment encoding a 3-repeat-FLAG epitope was cloned 5′ to the *Zfp36l1* cDNA to generate pcDNA3.1-3xFLAG-ZFP36L1 which expresses FLAG-tagged ZFP36L1 proteins. pGL3-*Pparγ*-luc, a luciferase reporter driven by *Pparγ* promoter was constructed as described previously [4].

### Lentivirus preparation and infection

For ZFP36L1 overexpression or knockdown in MC3T3-E1 cells, pLAS2w-ZFP36L1 or pshRNAZFP36L1 was cotransfected with gag-pol and VSV-G-expressing plasmids into 293T cells for the virus preparation as described previously [4]. Cells were infected with virus (MOI = 20) for 24 h in the presence of polybrene (8 μg/ml). Infected cells were selected with 2 μg/ml of puromycin (Enzo Life Sciences, Switzerland). Puromycin-resistent cells were pooled for experiments. For ZFP36L1 knockdown in C3H10T1/2 cells, infected cells were selected with 5 μg/ml of puromycin. Clone 6 which expressed the lowest level of *Zfp36l1* mRNA was selected for experiments.

### Cell culture

C3H10T1/2, MC3T3-E1, and 3T3-L1 cells were purchased from American Type Culture Collection. C3H10T1/2 and 3T3-L1 cells were maintained in DMEM (GIBCO-BRL, CA, USA), whereas MC3T3-E1 cells were maintained in MEMα (catalog no. A1049001, GIBCO-BRL, CA, USA). Culture media were supplemented with 10% fetal bovine serum, glutamine, penicillin, and streptomycin. Cells were cultured at 37 °C in a humidified atmosphere containing 5% CO2. C3H10T1/2 cells were transfected with pcDNA3.1-ZFP36L1 and selected with G418 (1 mg/ml). Twenty two antibiotic-resistant clones were harvested and examined for ZFP36L1 overexpression. Clones 2 and 21 which expressed high level of ZFP36L1 were selected for experiments. The femurs and bone marrow mesenchymal stem cells isolated from the femurs of adult and aged Fisher 344 rats were kind gifts from Dr. Chun-Chin Liang. For the isolation of RNA from rat femurs, the femurs were cryogenically pulverized at -195 °C by liquid N2 using a SPEX 6770 Freezer/Mill (SPEX SamplePrep, NJ, USA). Total RNA was then extracted using Trizol Reagent (Life Technology, MD, USA).

### Induction of osteoblastic and adipogenic differentiation

Osteoblastic induction on MC3T3-E1 and C3H10T1/2 cells, and adipogenic induction on 3T3-L1 and C3H10T1/2 cells were performed as described previously [4]. At the end of experiments, for osteoblastic differentiation, cells were fixed and stained with 2% Alizarin Red S solution (Sigma-Aldrich, MO, USA) for 30 min at room temperature, and were de-stained with freshly prepared 10% Cetylpyridinium chloride (CPC) (Sigma-Aldrich, MO, USA) solution for 1 hour at room temperature with gentle rocking. The CPC solutions were then collected for the measurement of absorbance at 595 nm. For adipogenic differentiation, cells were fixed and stained with 0.3% Oil Red O (Sigma-Aldrich, MO, USA), and were de-stained with isopropanol for the measurement of absorbance at 510 nm.

### Quantitative real-time PCR (RT-qPCR) and western blot analyses

Total RNA isolation, RT-qPCR analyses, and Western blot analyses were performed as described previously [4]. The 5′ and 3′ primers used were as follows: mouse *Zfp36l1*, CCCGATGGCACCAATAACC and GCCCCATGCTAGGAGCAA; mouse *Runx2*, CTCCGCTGTGAAAAACC and TGAAACTCTTGCCTCGTCC; mouse osteopontin, CCATCTCAGAAGCAGAATCTCC and ATGGTCATCATCGTCGTCC; mouse osteocalcin, TCTCTCTGACCTCACAGATCCC and TACCTTATTGCCCTCCTGCTTG; mouse *Hdac3*, CAGAGAGTCAGCCCCACCAA and GTAGACCACCGGCCCAGTT; mouse *Hdac4*, GGGAATGTACGACGCCAAAG and GCTGCATGCGGAGTCTGTAA; mouse *Hdac7*, GCTGGGTGGCTACCATGTTT and CTGAGGTTGGGTTTCTGTTTCC; mouse *Pparγ2*, TCGCTGATGCACTGCCTATG and GAGAGGTCCACAGAGCTGATT; mouse *aP2*, AAGAGAAAACGAGATGGTGACAA and CTTGTGGAAGTCACGCCTTT; mouse adiponectin, AGCCGCTTATATGTATCGCTCA and TGCCGTCATAATGATTCTGTTGG and mouse β-actin, CCCTGGCACCCAGCAC and GCCGATCCACACGGAGTAC. The antibodies used were anti-ZFP36L1 (Santa Cruz, CA, USA) and anti-β-actin (BD Biosciences, CA, USA).

### *Ex vivo* experiments and histological analysis

Cells were seeded into gelatin/EDC scaffolds and implanted subcutaneously into the back of male nude mice (8 weeks old) as described previously [4]. Implants were retrieved two weeks and one month later for the examination of adipogenic and osteoblastic differentiation by histological analysis, respectively. Histological sections were prepared from each implant and stained with either Oil Red O or Alizarin Red S and DAPI.

### Luciferase assays

To examine the influence of ZFP36L1 overexpression on the activity of *Pparγ* promoter, ZFP36L1-overexpressing and control C3H10T1/2 cells (1 × 10^6^) were transfected with 8 μg pGL3-*Pparγ*-luc together with a Renilla luciferase reporter (0.1 μg) as a normalizer. Cells were induced to undergo adipogenic differentiation, and were harvested at the times indicated for luciferase assays using the Dual-Luciferase Reporter Assay System (Promega, WI, USA).

### Preparation of biotin-labeled RNA transcripts and pull-down assays

Total RNA prepared from C3H10T1/2 cells was used to generate the 3′UTR of *Pparγ2* cDNA. A T7 RNA polymerase promoter sequence [19] was placed 5′ to the cDNA. The 5′ and 3′ primers used were as follows: (T7)CAGGAAAGTCCCACCCGC and AATTTTATAATGTGGTAATTTTTAAT. PCR-amplified products were purified. *In vitro* transcription was performed to generate biotin-labeled and unlabeled transcripts using the MegaScript T7 kit (ThermoFisher Scientific, MA, USA) following the manufacturer's instruction. The transcripts were purified, and 1 μg of biotin-labeled transcript was incubated with 150 μg of cytoplasmic lysates for 30 min at room temperature. The mixtures were then mixed with streptavidin-coated magnetic Dynabeads (ThermoFisher Scientific, MA, USA) to collect the biotin-linked ribonucleoprotein complexes. Western blot analyses were then performed to identify the protein component of the complexes.

### RNA electrophoretic mobility-shift assays (RNA EMSA)

Four-microgram aliquots of cytoplasmic fractions were incubated with 0.5 nM of biotin-labeled RNA probes and with certain amounts of unlabeled probes (for competition experiments) for 30 min at room temperature in a 20-μl mixture. The mixtures were electrophoresed through 4% nondenaturing polyacrylamide gels, and electrotransferred onto positively charged nylon membranes. Protein-RNA crosslink on the membranes was performed by exposing the membranes to UV using a Stratalinker (Stratagene, USA). The membranes were incubated with streptavidin-HRP (0.1 μg/ml), and the signals were visualized by the method as described for Western blot analyses.

### Immunoprecipitation of ribonucleoprotein complexes

Examination of the putative binding of ZFP36L1-containing protein complexes on *Pparγ2* mRNA was performed as described previously [19]. Briefly, ZFP36L1-overexpressing and control cells were mixed with equal volume of polysome lysis buffer plus inhibitors of RNases and proteases. The mixtures were centrifuged and the pellets were discarded. The supernatants were mixed for 16 h at 4°C with protein A beads pre-coated with either anti-ZFP36L1 or anti-IgG antibody. The beads were then washed with NT-2 buffer, and the RNAs were isolated from the precipitated ribonucleoprotein complexes for RT-qPCR analyses.

### Statistical analysis

Statistical difference was determined using Student's *t* test. Paired *t* test was used in *ex vivo* experiments.

## SUPPLEMENTARY MATERIALS FIGURES AND TABLES


