# Supplementary material for: Zinc finger protein ZFP36L1 promotes osteoblastic differentiation but represses adipogenic differentiation of mouse multipotent cells
Source: Oncotarget. 2017 Feb 9;8(13):20588–601. doi: 10.18632/oncotarget.15246 (PMC5400528; doi:10.18632/oncotarget.15246)
Supplement: Supplementary file 1 [file oncotarget-08-20588-s001.pdf]

Zinc finger protein ZFP36L1 promotes osteoblastic differentiation but represses adipogenic differentiation of mouse multipotent cells

Supplementary Material

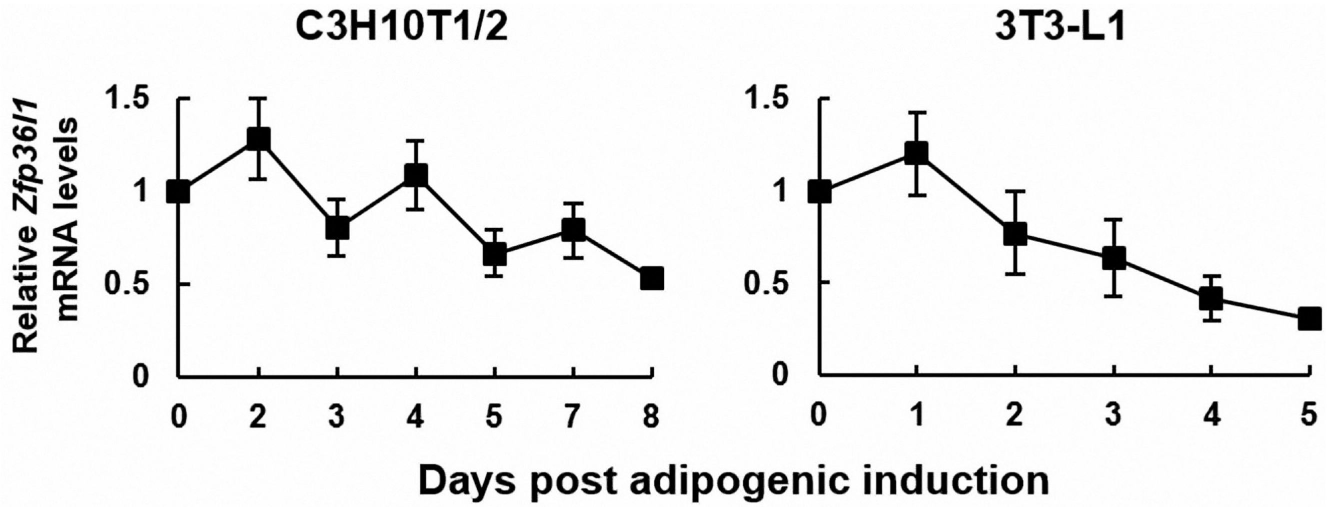

Figure S1: Kinetic expression of Zfp36l1 mRNA in C3H10T1/2 and 3T3-L1 cells under adipogenic induction. RT-qPCR analyses. Parental C3H10T1/2 and 3T3-L1 cells were induced to undergo adipogenic differentiation, and were harvested at the days as indicated for the measurement of *Zfp36l1* mRNA. Relative expression levels were calculated in relation to the control at day 0 (to which a value of 1 was assigned). Data represent the means ± S.D. from three experiments.

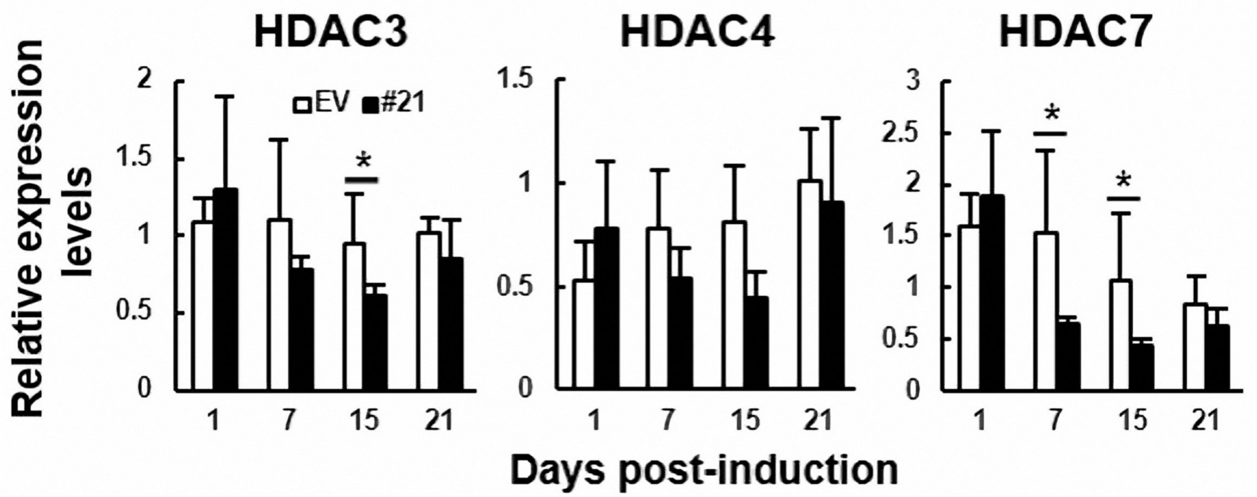

Figure S2: ZFP36L1 overexpression down-regulated the expression of Hdac3 and Hdac7 mRNAs. RT-qPCR analyses. C3H-EV (EV) and #21 cells were induced to undergo osteoblastic differentiation. Cells were harvested at times as indicated for measuring expression of *Hdac3*, *Hdac4*, and *Hdac7* mRNAs. The relative expression levels were obtained by comparing the mRNA levels of control and #21 cells to that of control cells of day 0 (to which a value of 1 was assigned). Data represent the mean ± S.D. from three experiments. *, P<0.05. The results showed that *HDAC3* expression appeared not to change with time in control cells, whereas its expression seemed to decrease in #21 cells; there was a significant difference between control and #21 cells on day 15. *HDAC4* expression in control and #21 cells seemed to change with time in different patterns; however, the difference did not reach statistical significance. *HDAC7* expression in control and #21 cells was decreased with time; there was a significant difference between control and #21 cells on day 7 and day 15 post induction. Our data, therefore, showed that ZFP36L1 overexpression down-regulated the expression of HDACs 3 and 7.

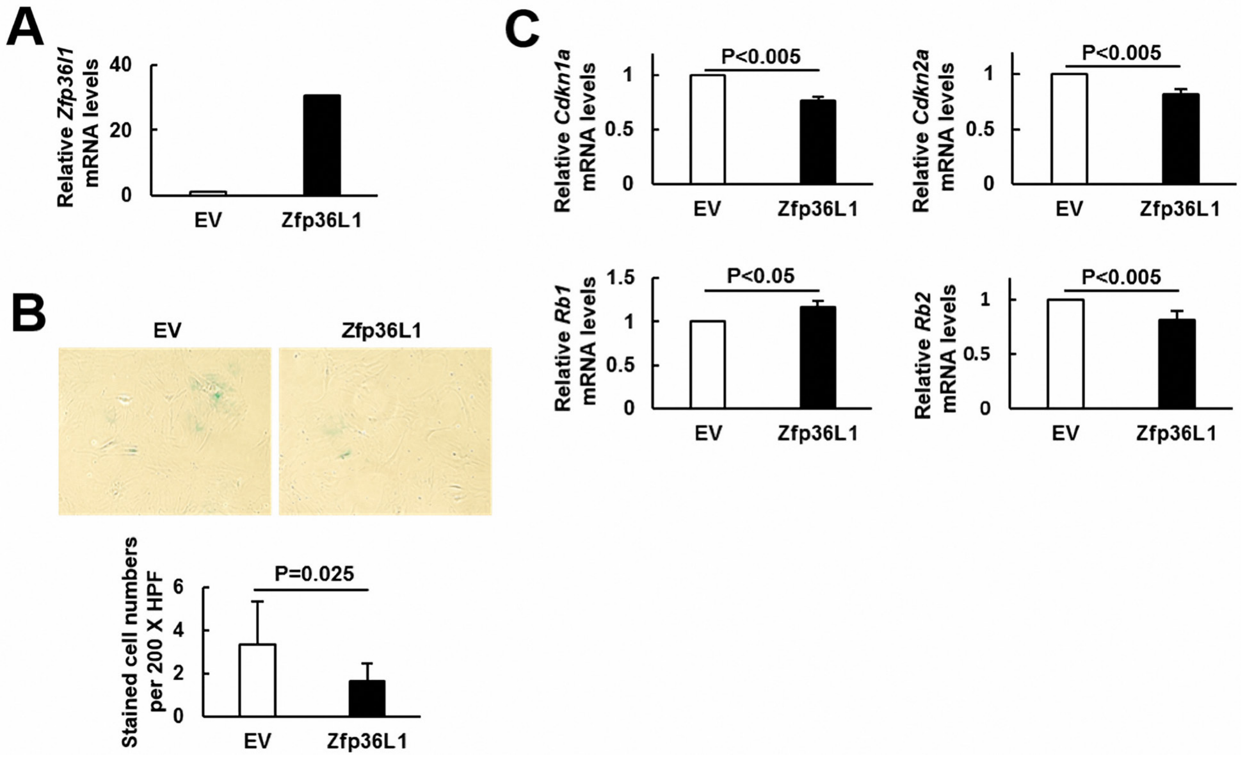

Figure S3: The effect of Zfp36L1 overexpression on the senescence phenotype of bmMSCs from aged rats. (A) RT-qPCR analyses. BmMSCs derived from aged F344 rats (20-month old) were used to establish the Zfp36L1-overexpressing (Zfp36L1) and control (EV) cells. The relative *Zfp36l1* mRNA levels are shown. (B) Staining of the cellular senescence-associated β-galactosidase. EV and Zfp36L1 cells were stained for the senescence-associated β-galactosidase using the Senescence Detection Kit (abcam, USA). The stained cells were counted in 8 fields under a 200 x high power field (HPF). Representative photos are shown. Data represents the mean ± S.D. (n=8). (C) RT-q-PCR analyses. The expression of *Cdkn1a*, *Cdkn2a*, *Rb1*, and *Rb2* mRNAs in EV and Zfp36L1 cells were examined. The relative expression levels were obtained by comparing the mRNA levels of Zfp36L1 cells to that of control cells (to which a value of 1 was assigned). Data represent the mean ± S.D. from three experiments.
